# Carbamylated albumin is one of the target antigens of anti-carbamylated protein antibodies

**DOI:** 10.1093/rheumatology/kex088

**Published:** 2017-04-07

**Authors:** Shuichiro Nakabo, Motomu Hashimoto, Shinji Ito, Moritoshi Furu, Hiromu Ito, Takao Fujii, Hajime Yoshifuji, Yoshitaka Imura, Ran Nakashima, Kosaku Murakami, Nobuo Kuramoto, Masao Tanaka, Junko Satoh, Akihito Ishigami, Satoshi Morita, Tsuneyo Mimori, Koichiro Ohmura

**Affiliations:** 1 Department of Rheumatology and Clinical Immunology; 2 Department of the Control for Rheumatic Diseases; 3 Medical Research Support Center; 4 Department of Orthopaedic Surgery, Graduate School of Medicine, Kyoto University, Kyoto; 5 Department of Clinical Immunology and Rheumatology, Wakayama Medical University, Wakayama; 6 Tokyo Metropolitan Institute of Gerontology, Molecular Regulation of Aging, Tokyo; 7 Department of Biomedical Statistics and Bioinformatics, Graduate School of Medicine, Kyoto University, Kyoto, Japan

**Keywords:** rheumatoid arthritis, autoantigens, autoantibodies, laboratory diagnosis, neutrophils

## Abstract

**Objectives.** Anti-carbamylated protein (anti-CarP) antibodies are detected in RA patients. Fetal calf serum is used as an antigen source in anti-CarP ELISA, and the precise target antigens have not been found. We aimed to identify the target antigens of anti-CarP antibodies.

**Methods.** Western blotting of anti-CarP antibodies was conducted. Anti-carbamylated human albumin (CarALB) antibody was detected by in-house ELISA for 493 RA patients and 144 healthy controls (HCs). An inhibition ELISA of anti-CarP antibodies by CarALB and citrullinated albumin (citALB) was performed using eight RA patients’ sera. Serum CarALB was detected by liquid chromatography–tandem mass spectroscopy (LC/MS/MS), and the serum MPO concentration was measured by ELISA.

**Results.** We focused on carbamylated albumin because it corresponded to the size of the thickest band detected by western blotting of anti-CarP antibodies. Anti-CarALB antibody was detected in 31.4% of RA patients, and the correlation of the titres between anti-CarALB and anti-CarP was much closer than that between anti-citALB and anti-CCP antibodies (ρ = 0.59 and ρ = 0.16, respectively). The inhibition ELISA showed that anti-CarP antibodies were inhibited by CarALB, but not by citALB. CarALB was detected in sera from RA patients by LC/MS/MS. The serum MPO concentration was correlated with disease activity and was higher in RA patients with anti-CarALB antibody than in those without.

**Conclusion.** We found that carbamylated albumin is a novel target antigen of anti-CarP antibodies, and it is the first reported target antigen that has not been reported as the target of ACPA.

Rheumatology key messages

- Anti-carbamylated human albumin antibody is detectable in 31.4% of RA patients.
- Anti-carbamylated human albumin antibody is not the cross-reaction of ACPA.
- The positivity of anti-carbamylated human albumin antibody was associated with high serum MPO level.

## Introduction

Carbamylation is a post-translational modification of proteins induced by cyanate, and is the non-enzymatic and irreversible conversion of lysine into homocitrulline [1]. Cyanate exists in equilibrium with urea in the blood and can be produced from thiocyanate by MPO [2]. Therefore, carbamylation occurs not only in uraemic patients [3–5], but also at chronic inflammatory sites, including those in atherosclerosis [2].

Recently, anti-carbamylated protein (anti-CarP) antibodies were reported to be new autoantibodies of RA [6]. They have drawn attention as new diagnostic biomarkers because they can be detected in RA patients without ACPA. Moreover, they have also been reported to be associated with disease severity [6, 7] and detected before the onset of RA development [8–10].

However, the precise target antigen of anti-CarP antibodies has not been elucidated. Anti-CarP antibodies are detected by ELISA and western blotting, in which carbamylated fetal calf serum (FCS) is used as a complex of the protein mixture [6]. Fibrinogen and vimentin have been reported as target antigens [6, 11–13], but they are also known as the target antigens of ACPA. Given that homocitrulline closely resembles citrulline, the cross-reaction between anti-CarP antibodies and ACPA has been a concern. In fact, anti-CarP antibodies and ACPA frequently coexist [6, 14] and show cross-reaction to some extent [15]. 

Therefore, it is desirable to find new target antigens of anti-CarP antibodies that are not targets of ACPA in order to resolve the concern regarding cross-reaction and evaluate the role of anti-CarP antibodies.

In this study, we focused on carbamylated human albumin (CarALB) as a target antigen of anti-CarP antibodies and attempted to identify the factors that are related to anti-CarALB antibody.

## Methods

### Patients and clinical information

A total of 493 RA patients were recruited from Kyoto University Hospital, and their clinical information was obtained from the Kyoto University Rheumatoid Arthritis Management Alliance (KURAMA) database, which was established in 2011 and contains detailed clinical information added yearly. One hundred and forty-four control sera being unlinkably anonymized were obtained from healthy donors. Written informed consent was obtained from the participants to enrol in this database. All the data were analysed anonymously. This study was designed in accordance with the Declaration of Helsinki and was approved by Kyoto University Graduate School and Faculty of Medicine, Ethics Committee (approval number: E1308).

### Protein preparation

FCS (Biowest, Nuaillé, France), BSA (Sigma-Aldrich, St Louis, MO, USA) and human albumin (ALB; Sigma-Aldrich) were carbamylated by potassium cyanate (KOCN; Nacalai Tesque, Kyoto, Japan) following the method previously reported [6, 16]. In short, equal volumes of 2 mol/l of KOCN in distilled water and 4 mg/ml of FCS, 2 mg/ml of BSA or 2 mg/ml of ALB were mixed and incubated overnight at 37 °C.

ALB was also citrullinated by incubation with rabbit skeletal peptidylarginine deiminase (Sigma-Aldrich) for 3 h at 50°C, as previously described [17].

Successful protein carbamylation was confirmed by mass spectrometry or western blotting using rabbit anti-carbamyl-lysine polyclonal antibody (anti-CBL antibody; Cell Biolabs, San Diego, CA, USA). Citrullination was confirmed by the anti-modified citrulline-Senshu method [18].

### Western blotting

Carbamylated FCS (CarFCS) and unmodified FCS (UmFCS), or carbamylated BSA (CarBSA) and unmodified BSA (UmBSA) were electrophoresed and transferred onto nitrocellulose membranes. Membranes were incubated for 5 min in bullet blocking one for western blotting (Nacalai Tesque) or for 30 min in 5% skimmed milk in PBS with 0.05% Tween 20 (PBS-T); the former was for sera from patients and the latter for anti-BSA antibody, described below.

The membranes were incubated for 1 h in sera from patients or rabbit anti-BSA antibody (Sigma-Aldrich) diluted 1000-fold by each blocking buffer followed by three washes in PBS-T. Sera were obtained from seven RA patients with anti-CarP antibodies. Subsequently, they were incubated for 1 h in horseradish peroxidase-conjugated anti-human or anti-rabbit IgG (Promega, Madison, WI, USA) diluted 10 000-fold by each blocking buffer. Finally, they were washed three times, and antibodies were detected by SuperSignal™ West Pico Chemiluminescent Substrate (Thermo Fisher Scientific, Waltham, MA, USA). All the procedures were conducted at room temperature.

### ELISA

We established an in-house anti-CarP-antibody ELISA system following the original report [6] with a few modifications. UmFCS or CarFCS was diluted to 0.02 mg/ml with pH 9.6 0.1 mol/l carbonate/bicarbonate buffer and then used to coat Nunc Maxisorp plates (Thermo Fisher Scientific) at a volume of 50 μl overnight at 4 °C. Then the plates were washed three times in PBS-T followed by blocking with PBS containing 2% BSA (BSA-PBS) for 6h at 4 °C. All the washing procedures were conducted three times using PBS-T. Next, sera from patients (150-fold dilution by BSA–PBS) were applied to the ELISA plates in 50 μl. After overnight incubation at 4 °C and washing, the wells were reacted for 3 h with 50 μl of rabbit anti-human IgG antibody (Thermo Fisher Scientific), which was diluted 10 000-fold with BSA–PBS. After washing, 50 μl of alkaline phosphatase-conjugated anti-rabbit IgG (Promega) diluted 2000-fold with BSA–PBS was added and incubated for 3 h at 4 °C followed by washing, and finally 3,3-diaminobenzidine (Sigma-Aldrich) was added. The optical density of each well was read at 405 nm.

A standard curve was drawn using serum from one patient as standard. Arbitrary units of the anti-CarP titre were calculated by subtracting the titre against UmFCS from that against CarFCS. Negative values were regarded as zero. The cut-off value was set at the titre that makes the specificity higher than 0.95 based on the analysis of the receiver operating characteristic curve of our data.

Anti-CarALB and anti-citrullinated ALB (anti-citALB) antibody ELISA systems were established by modifying the anti-CarP-antibody ELISA. Points differing from the anti-CarP-antibody ELISA were as follows. Although the anti-CarP ELISA consisted of three types of antibodies (primary, secondary and labelled tertiary), the anti-CarALB and anti-citALB ELISAs involved two steps (primary and labelled secondary antibodies). The secondary antibody was alkaline phosphatase-conjugated anti-human IgG (Promega). After the blocking step, each procedure was performed at room temperature. Reaction steps for patients’ sera and anti-human IgG were for 2 and 1.5 h, respectively.

### Inhibition assay

We chose eight strongly anti-CarP-positive patients’ sera and incubated them overnight at 4 °C in each concentration of UmFCS (negative control), CarFCS (positive control), citALB or CarALB. Subsequently, they were analysed by the anti-CarP-antibody ELISA system.

In the same manner, inhibition assays of anti-CarALB and anti-citALB antibodies were performed using eight other RA patients’ sera with both anti-CarALB and anti-citALB antibodies. They were incubated overnight at 4 °C in each concentration of CarALB, citALB or unmodified ALB (UmALB).

### Calculation of carbamylation index of serum albumin based on data obtained by liquid chromatography–tandem mass spectrometry

Sera from four strongly anti-CarALB-antibody-positive RA patients, three anti-CarALB-antibody-negative RA patients, three healthy controls (HCs) and a chronic kidney disease (CKD) patient were diluted, electrophoresed (SDS–PAGE) and dyed by Rapid Stain CBB Kit (Nacalai Tesque). Purified albumin was recovered from gel pieces corresponding to the band for albumin. The in-gel digestion and extraction of albumin peptides were carried out using In-Gel Tryptic Digestion Kit (Thermo Scientific) according to the manufacturer’s instructions.

The tryptic digests were resuspended in 0.1% formic acid and separated using Nano-LC-Ultra 2 D-plus equipped with cHiPLC Nanoflex (Eksigent, Dublin, CA, USA) in the trap-and-elute mode, with a trap column [200 μm × 0.5 mm ChromXP C18-CL 3 μm 120 Å (Eksigent)] and an analytical column [75 μm × 15 cm ChromXP C18-CL 3 μm 120 Å (Eksigent)]. The separation was carried out using a binary gradient with solvent A (98% water, 2% acetonitrile and 0.1% formic acid) and solvent B (20% water, 80% acetonitrile and 0.1% formic acid). The gradient programme was as follows: 2–40% of solvent B for 125 min, 90% of solvent B for 5 min and 2% of solvent B for 19 min, at a flow rate of 300 nl/min. The eluates from the analytical column were directly infused into a mass spectrometer (TripleTOF 5600+ System with NanoSpray III source and heated interface; SCIEX, Framingham, MA, USA).

Data acquisition was carried out with an information-dependent acquisition method, and the acquired data sets were imported to the platform of Progenesis QI for proteomics software version 2.0 (Nonlinear Dynamics, Newcastle upon Tyne, UK) for label-free, global quantification of peptides. The tandem mass spectrometry (MS/MS) spectrum corresponding to the peaks found by Progenesis QI for proteomics were exported for identification by Mascot Server version 2.4.1 (Matrix Science, London, UK) with SwissProt database for human (July 2016). Carbamidomethyl (C) as fixed modification, and Oxidation (M), acetyl (protein N-term), carbamyl (K) and carbamyl (N-term) as variable modifications, were taken into consideration. The relative abundance of each peptide was calculated using the method to normalize to all proteins provided by the Progenesis QI for proteomics software.

The carbamylation index was calculated using the following equation: carbamylation index = sum of normalized abundance of peptide whose lysine is carbamylated/sum of normalized abundance of peptide containing lysine residues. Peptides with Mascot peptide scores above the identification level were used for this calculation. All liquid chromatography (LC)-MS/MS analyses were performed twice in differently randomized injection orders, and the mean carbamylation index was calculated based on the average of the two trials for each sample.

### Analysis of serum MPO

Sera from 70 RA patients whose titres of the anti-CarALB antibody were the first to the 35th highest and the first to the 35th lowest, and sera from 11 HCs were chosen to test the serum MPO concentration. Serum MPO was quantified using Quantikine ELISA Human Myeloperoxidase Immunoassay (R&D Systems, Minneapolis, MN, USA) following the manufacturer’s instructions.

### Statistical analysis

All statistical analyses were conducted using R version 3.1.1 [19]. Correlations between the titres of each antibody were analysed using Spearman’s rank correlation coefficient. Significance was calculated by the Mann–Whitney *U*-test with Bonferroni’s correction in MPO ELISA. Correlations between the MPO titre and DAS28 were analysed using the Pearson product–moment correlation coefficient. On clinical evaluation, the Mann–Whitney *U*-test was used for continuous variables and the χ^2^ test or Fisher’s exact test was used for categorical variables. Binary logistic regression was used to assess odds ratios and 95% CIs for the presence of the anti-CarALB antibody. Under the assumption that the missing data occurred completely at random, pairwise and listwise deletions were used in uni- and multivariate analyses, respectively.

## Results

**Fig. 1:**
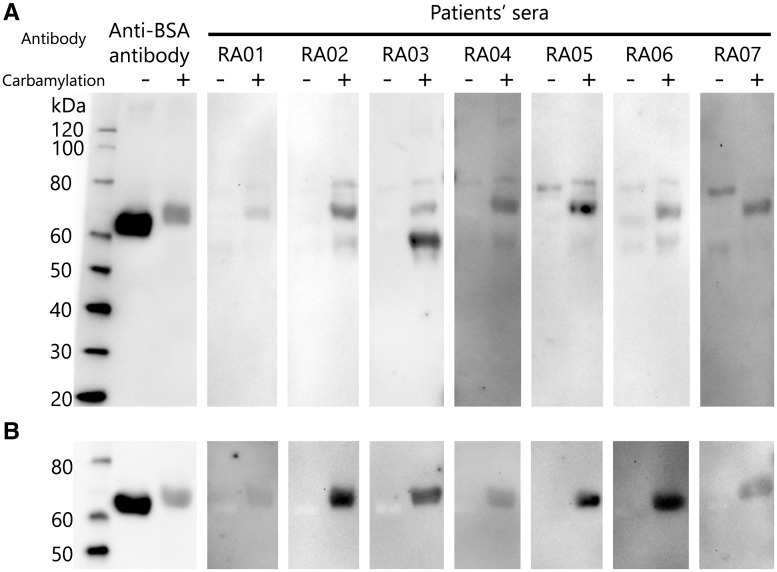
Western blot analysis of anti-carbamylated protein antibodies Carbamylated and unmodified fetal calf serum (FCS) samples were electrophoresed and blotted onto a nitrocellulose membrane, and then incubated with sera from seven RA patients containing a high titre of anti-carbamylated protein (anti-CarP) antibodies or anti-BSA polyclonal antibody. (**A**) The common band detected in sera from RA patients corresponded to carbamylated albumin. (**B**) Western blotting was also performed using unmodified and carbamylated BSA.

In order to identify the target antigens of anti-CarP antibodies, we first performed western blotting. UmFCS and CarFCS were blotted onto the membrane and then incubated with sera containing high titres of anti-CarP antibodies from seven RA patients. The band commonly detected in the seven RA patients’ sera corresponded to a 70 kDa protein (Fig. 1A). As the size of the bands was similar to that of albumin detected by anti-BSA antibody and albumin was also the most abundant protein in FCS, we considered that the main target antigen of anti-CarP antibodies was albumin. As expected, the western blotting using unmodified BSA (UmBSA) and carbamylated BSA (CarBSA) showed similar results (Fig. 1B). These results strongly suggest that the antibody against CarBSA is a major component of anti-CarP antibodies.

**Fig. 2:**
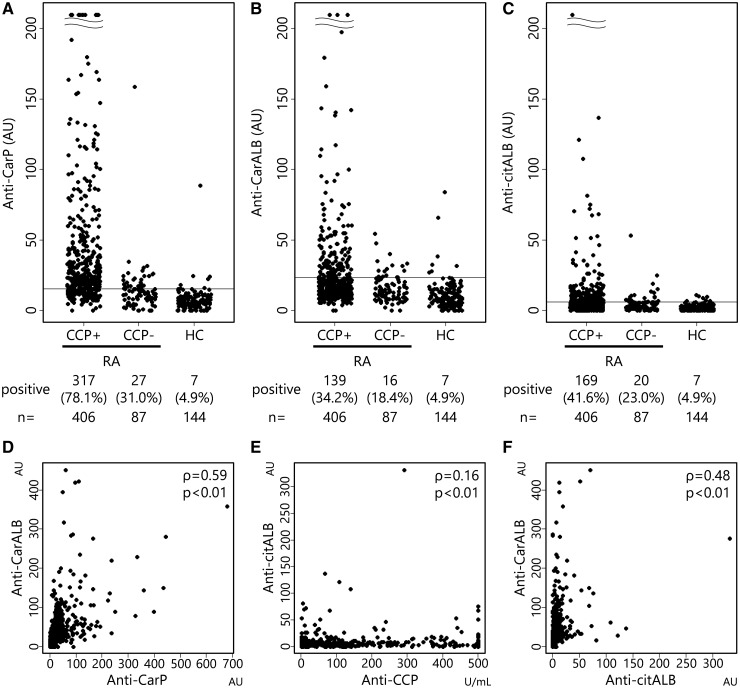
Positivity and correlation of anti-carbamylated protein, anti-carbamylated albumin and anti-citrullinated albumin antibodies in RA patients ELISAs of anti-carbamylated protein (anti-CarP) antibody (**A**), anti-carbamylated human albumin (anti-CarALB) antibody (**B**) and anti-citrullinated human albumin (anti-citALB) antibody (**C**) were performed, and titres of these antibodies are plotted in anti-CCP antibody-positive RA patients, anti-CCP antibody-negative RA patients and healthy controls (HCs). Horizontal lines represent cut-off values for each antibody, set by the data from HCs. Correlations of antibody titres in RA patients are shown by the plots between anti-CarP and anti-CarALB antibodies (**D**), between anti-CCP and anti-citALB antibodies (**E**) and between anti-CarALB and anti-citALB antibodies (**F**). Spearman’s rank correlation coefficient (ρ) was used to identify the relationships between them.

We next established in-house ELISA systems for anti-CarALB, anti-citALB and anti-CarP antibodies. Sera from 493 RA patients and 144 HCs were assayed. Anti-CarP was positive in 344 RA patients (69.8%). When stratified by the positivity of anti-CCP antibody, anti-CarP antibody was mainly detected in CCP-positive RA (Fig. 2A), which is consistent with previous reports [6, 14]. Anti-CarALB antibody was detected in 155 RA patients (31.4%), and was also detected mainly in the CCP-positive RA group (Fig. 2B). Sensitivities of anti-CarALB in CCP-positive RA and CCP-negative RA were 34.2 and 18.4%, respectively. In cotrast, the prevalence of anti-citALB antibody was comparable to that of anti-CarALB antibody (38.3%; Fig. 2C).

Next, we assessed the correlation of anti-CarALB and anti-CarP titres. They showed a good correlation (ρ = 0.59; Fig. 2D), suggesting that albumin is the major constituent of target antigens of anti-CarP antibodies. In contrast, anti-citALB and anti-CCP titres showed almost no correlation (ρ = 0.16; Fig. 2E), suggesting that albumin is not the target antigen of ACPAs. The correlation of anti-CarALB and anti-citALB titres was modest (ρ = 0.48; Fig. 2F), which may indicate that anti-CarALB antibody cross-reacts with citALB to some extent.

**Fig. 3:**
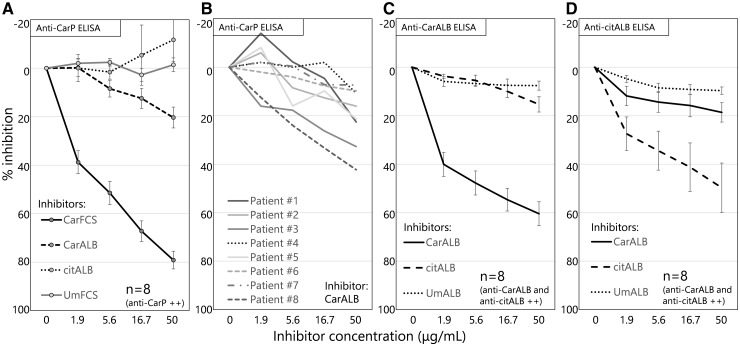
Inhibition ELISA of anti-carbamylated protein antibodies, anti-carbamylated albumin antibody and anti-citrullinated albumin antibody Anti-carbamylated protein (Anti-CarP) antibodies were inhibited by different concentrations of carbamylated fetal calf serum (CarFCS), carbamylated albumin (CarALB), citrullinated albumin (citALB) or unmodified FCS (UmFCS). (**A**) Percentage inhibitions of the anti-CarP titre were plotted using sera containing a high titre of anti-CarP antibodies from eight RA patients. Data represent the means (s.e.m.) (**B**) Percentage inhibition of anti-CarP antibody by CarALB exhibited by sera from individual patients. In a similar way, anti-CarALB antibody (**C**) and anti-citALB antibody (**D**) were inhibited by different concentrations of CarALB, citALB and unmodified albumin (UmALB). The sera used were from eight RA patients with both anti-CarALB and anti-citALB antibodies.

In order to clarify that CarALB and not citALB is the target antigen of anti-CarP antibody, we performed an inhibition ELISA assay. Sera with high titres of anti-CarP antibody from eight RA patients were pre-incubated with different concentrations of CarFCS, CarALB, citALB or UmFCS and then titres of anti-CarP antibody were measured. The titre of anti-CarP antibody was mildly inhibited by CarALB, but was not inhibited by citALB at all (Fig. 3A). Although the strength of the inhibition by CarALB varied among patients, all the samples were inhibited by CarALB to some extent (Fig. 3B), which indicates that CarALB is one of the target antigens of anti-CarP antibodies and that the repertoire of target antigens is different among patients.

Next, we assessed the cross-reactivity between anti-CarALB and anti-citALB antibodies using a similar inhibition ELISA. Sera from eight RA patients with both anti-CarALB and anti-citALB antibodies were pre-incubated with different concentration of CarALB, citALB and UmALB and then titres of anti-CarALB and anti-citALB antibodies were measured. The anti-CarALB and anti-citALB titres were hardly affected by citALB and CarALB, respectively (Fig. 3C and D). This shows that cross-reactions between anti-CarALB and anti-citALB antibodies are limited.

We then performed LC-MS/MS in order to clarify whether serum albumin is carbamylated in RA patients. Sera from four RA patients with anti-CarALB antibody, three RA patients without anti-CarALB antibody, three HCs and a CKD patient were examined. The CKD patient’s serum was regarded as the positive control because the proportion of carbamylated albumin has been reported to be high in end-stage renal disease patients [20]. The carbamylation index was calculated based on the abundance of albumin peptides with homocitrulline over those with lysine. As shown in Table 1, we could detect some carbamylation of albumin even in the healthy people. The carbamylation index of anti-CarALB-positive RA patients was slightly higher than that of anti-CarALB-negative RA patients or HCs, but the differences were modest compared with the CKD patients. Although the interpretation of these data requires further investigation, at least we can conclude that carbamylation of albumin occurs *in vivo*.

**Table 1:** Background information and carbamylation index of patients and healthy subjects

Sample number	1	2	3	4	5	6	7	8	9	10	11
Disease	RA	RA	RA	RA	RA	RA	RA	HC	HC	HC	CKD
Anti-CarALB antibody	+	+	+	+	−	−	−	−	−	−	NA
(titre, a.u.)	(179.3)	(142.2)	(143.6)	(225.7)	(6.2)	(0.0)	(8.5)	(21.2)	(9.8)	(7.2)	
DAS28 (ESR)	5.4	5.1	3.5	5.4	NA	2.5	4.6	NA	NA	NA	NA
CRP, mg/dl	1.3	0	0.2	2.8	0.1	0.1	23.8	NA	NA	NA	0.1
BUN, mg/dl	14	16	29	11	15	18	12	NA	NA	NA	64
CRE, mg/dl	0.73	0.52	0.93	0.51	0.6	0.6	0.48	NA	NA	NA	5.87
Carbamylation index^a^	17.0	14.2	17.4	13.4	13.6	12.6	14.0	16.5	12.7	9.8	30.4

a Mean of carbamylation index scores from two independent experiments. Anti-CarALB: anti-carbamylated human albumin; a.u.: arbitrary units; BUN: blood urea nitrogen; carbamylation index: sum of normalized abundance of peptide whose lysine is carbamylated/sum of normalized abundance of peptide containing lysine residues; CKD: chronic kidney disease; CRE: serum creatinine; HC: healthy control; NA: not available.

Given that carbamylation is induced by cyanate that is produced from thiocyanate enzymatically with MPO *in vivo* [2], we speculated that serum albumin was carbamylated by MPO released from inflammatory sites. Therefore, we measured serum MPO concentrations in the sera from 35 RA patients with high titres of anti-CarALB antibody, 35 RA patients without anti-CarALB antibody and 11 HCs, using the MPO ELISA kit.

**Fig. 4:**
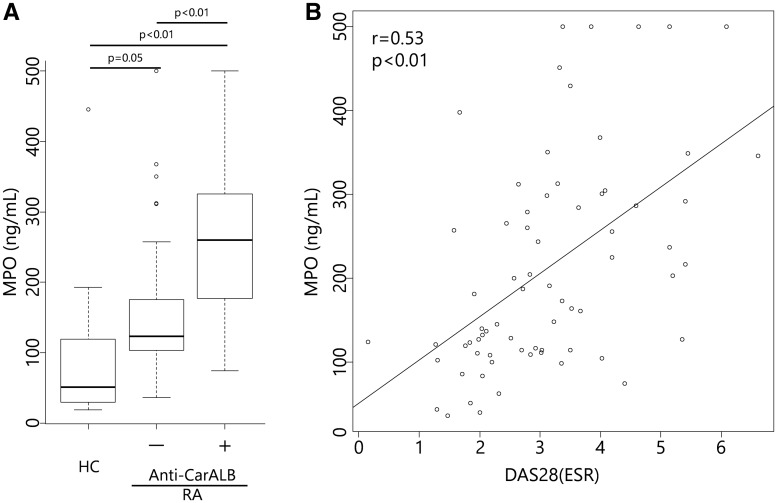
Serum MPO concentrations (**A**) Serum MPO concentrations of 35 RA patients with anti-carbamylated albumin (anti-CarALB) antibody, 35 RA patients without anti-CarALB antibody and 11 healthy controls. Serum MPO concentrations were significantly different between the groups. The median with interquartile range is shown. (**B**) The serum MPO concentration and DAS28 (ESR) in all RA patients showed a good correlation.

The serum MPO concentration was significantly higher in RA patients with anti-CarALB than in those without anti-CarALB and HCs (Fig. 4A). Notably, the serum MPO concentration was correlated with disease activity (Fig. 4B), supporting the idea that MPO released from inflammatory sites carbamylates serum albumin, which triggers anti-CarALB antibody production.

Finally, we assessed the clinical relevance of anti-CarALB using clinical data from the KURAMA cohort (Table 2). The presence of anti-CarALB antibody was significantly correlated with a higher Steinbrocker stage, higher DAS28, biologic use, as well as anti-CCP and anti-CarP antibodies. In multivariate analysis, DAS28, history of cardiovascular disease (CVD), blood urea nitrogen, MTX use, biologic use and anti-CarP antibodies were independently associated with the presence of anti-CarALB antibody, whereas the Steinbrocker stage and anti-CCP antibody were not (Table 2).

**Table 2:** Comparison of clinical characteristics between RA patients with and without anti-CarALB antibody

Covariate	**Anti-CarALB**		Univariate	**Multivariate**	
	**Positive, n = 155**	**Negative, n = 338**	**P-value**	**OR (95% CI)**	**P-value**
Age, mean (s.d.), years	62.8 (12.8)	62.2 (13.6)	0.85	1.00 (0.97, 1.02)	0.75
Disease duration, mean (s.d.), years	17.5 (19.7)	15.6 (18.3)	0.12	0.99 (0.97, 1.01)	0.48
Women, %	81.9	85.2	0.36	0.78 (0.55, 2.94)	0.57
Steinbrocker stage					
Low stage			0.01	1.49 (0.78, 2.93)	0.23
I	21	63			
II	29	82			
High stage					
III	30	50			
IV	66	114			
DAS28 (ESR), mean (s.d.)	3.24 (1.28)^a^	2.85 (1.12)^b^	<0.01	1.29 (1.02, 1.62)	0.03
Smoking			0.14	1.75 (0.94, 3.28)	<0.08
Current + former	18 + 34	26 + 62			
Never	80	187			
Cardiovascular disease, %	4.9^c^	2.3^d^	0.15	5.85 (1.27, 26.82)	0.02
BUN, mean (s.d.), mg/dl	15.9 (5.2)	16.2 (6.1)	0.94	0.94 (0.89, 0.99)	0.02
Anti-CCP antibody-positive, %	89.7	79.0	<0.01	1.21 (0.35, 1.94)	0.55
Anti-CarP antibody-positive, %	92.3	59.5	<0.01	9.12 (3.83, 21.7)	<0.01
MTX use, %	61.6^e^	70.1^f^	0.07	0.50 (0.28, 0.89)	0.02
Biologic use, %	41.1^g^	28.6^h^	<0.01	1.75 (1.01, 3.02)	0.05

Missing data are as follows: Steinbrocker stage, n = 38; DAS28 (ESR), n = 85; smoking, n = 86; cardiovascular disease, n = 42; MTX use, n = 36; and biologic use, n = 36. Pairwise deletion was used in univariate analysis. In contrast, 151 patients were excluded by listwise deletion in multivariate analysis.

a Data from 22 patients were not available.

b Data from 63 patients were not available.

c Data from 12 patients were not available.

d Data from 30 patients were not available.

e Data from nine patients were not available.

f Data from 27 patients were not available.

g Data from nine patients were not available.

h Data from 27 patients were not available. Anti-CarALB: anti-carbamylated human albumin; biologic: abatacept, adalimumab, certolizumab pegol, etanercept, golimumab, infliximab and tocilizumab; BUN: blood urea nitrogen.

## Discussion

In the present study, we showed that albumin is one of the target antigens of anti-CarP antibodies. This is the first target antigen that has not been reported as a target of ACPA. We also showed that CarALB exists in sera from RA patients. The serum MPO concentration was higher in RA patients with anti-CarALB antibody than in those without it, and the MPO concentration was clearly correlated with the disease activity. Furthermore, the presence of anti-CarALB antibody was associated with more severe disease activity. All these results suggest the involvement of anti-CarALB or anti-CarP antibodies in the pathophysiology of RA.

Previously reported target antigens of anti-CarP antibodies are fibrinogen and vimentin [6, 11–13], which are also known as target antigens of ACPA. As cross-reaction between anti-CarP antibody and ACPA has been a concern, the identification of antigens that are recognized only by anti-CarP antibodies has been desired. We showed that inhibition ELISA of anti-CarALB antibody by citALB hardly changed the titre of anti-CarALB antibodies, which indicates that anti-CarALB antibody is not the cross-reaction of ACPA and strongly supports the notion that anti-CarP antibody exists independently of ACPA.

The mechanism of anti-CarALB antibody production in RA has yet to be clarified. *In vivo*, protein carbamylation naturally occurs by cyanate, which exists in equilibrium with urea [21], as we found in HC sera (Table 1). However, cyanate is also synthesized from thiocyanate by MPO enzymatically at inflammatory sites [2]. The plasma thiocyanate concentration was reported to be higher in smokers [22], which is interesting because smoking is thought to be a risk factor for RA [23, 24]. Previous studies demonstrated that the serum or plasma MPO concentration was higher in patients with RA than patients with OA or HCs [25, 26], and a higher level of protein carbamylation was detected in the blood and synovial fluid of RA patients [27, 28]. These reports are also consistent with our results showing that the serum MPO concentration was correlated with disease activity (DAS28). Overall, the carbamylation of albumin occurs naturally, but is enhanced by MPO from inflammatory sites, including the RA synovium, which may trigger the disruption of tolerance to produce anti-CarALB antibody.

Currently, we do not have data on how the tolerance against carbamylated albumin is disrupted. However, Mydel *et al.* [27] reported that the severest arthritis was induced after the intra-articular injection of citrullinated peptides in mice immunized with carbamylated peptides or carbamylated mouse albumin. This suggests the immunodominance of carbamylated peptide and protein. The coexistence of carbamylated and citrullinated proteins may be important for the pathogenicity of RA. We believe that the key players are neutrophils, because MPO is an enzyme released from activated neutrophils, and neutrophil extracellular traps are thought to be important for ACPA induction [29–31]. The fact that anti-CarALB antibody frequently coexisted with anti-CCP antibody supports this idea. However, further research is required to clarify the relationship between neutrophils and anti-CarALB antibody.

In contrast, it is noteworthy that a correlation of anti-CarALB antibody with CVD incidence was shown. A high plasma homocitrulline concentration [2] and carbamylated low-density lipoprotein [32] were reported to be risk factors for CVD. It is also well known that the risk of CVD in RA patients is increased [33–35]. Although we did not examine whether other proteins are carbamylated, our data suggest that protein carbamylation is one reason for a high CVD risk in RA patients.

### Conclusions

We found that carbamylated albumin is a novel target antigen of anti-CarP antibodies, and it is the first reported target antigen that has not been reported as the target of ACPA.
